# Comparison of Novel Oral Anticoagulants and Vitamin K Antagonists in Patients With Cerebral Venous Sinus Thrombosis on Efficacy and Safety: A Systematic Review

**DOI:** 10.3389/fneur.2020.597623

**Published:** 2020-12-10

**Authors:** Hongjie Li, Meiling Yao, Songjie Liao, Jingyan Chen, Jian Yu

**Affiliations:** Guangdong Provincial Key Laboratory of Diagnosis and Treatment of Major Neurological Diseases, National Key Clinical Department and Key Discipline of Neurology, Department of Neurology, Guangdong Provincial Engineering Center For Major Neurological Disease Treatment, Guangdong Provincial Translational Medicine Innovation Platform for Diagnosis and Treatment of Major Neurological Disease, Guangdong Provincial Clinical Research Center for Neurological Diseases, The First Affiliated Hospital, Sun Yat-sen University, Guangzhou, China

**Keywords:** cerebral venous sinus thrombosis, novel oral anticoagulants, hemorrhage, efficacy, anticoagulant

## Abstract

Vitamin K antagonists (VKAs) are guideline-suggested subacute anticoagulants for cerebral venous sinus thrombosis (CVST), although there is potential hemorrhage risk in clinical use. In the last decade, novel oral anticoagulants (NOACs) have been applied as an alternative to VKAs in some kinds of thromboembolic diseases. Whether NOACs could replace VKAs in CVST treatment remains unclear. We conducted a comparison between the two types of medicines on efficacy and safety for the treatment of CVST based on the present clinical evidence from a literature search. Six studies [four retrospective studies, one prospective study, and 1 randomized clinical trial (RCT)] including 398 patients were included. Data suggested no significant difference between NOACs and VKAs in terms of recurrence of venous thrombotic events (VTEs) or death [risk ratio (RR) = 0.34, 95% confidence interval (CI) 0.06–1.98], partial recanalization (RR = 0.97, 95% CI 0.93–1.14), and overall hemorrhage events (RR = 0.86, 95% CI 0.47–1.58). In conclusion, the application of NOACs for CVST is similar to that of VKAs in terms of efficacy and safety.

## Introduction

Cerebral venous sinus thrombosis (CVST), characterized by thrombosis of the dural sinus or cerebral vein, is a rare type of stroke responsible for 0.5% of all strokes (1, 2). A few months after the acute phase of CVST, patients still suffer a high risk of venous thrombotic events (VTEs). The estimated recurrence rate of VTEs was reported from 2.0 to 4.1 per hundred persons per year, while that of CVST was in 1.5 per hundred persons per year (3, 4). For now, anticoagulant therapy has been recommended as the first-line therapy to prevent the recurrence, promote recanalization, and prevent early rethrombosis within a partially recanalized sinus of CVST and other VTEs (5). Specifically, warfarin, a kind of vitamin K antagonists (VKAs), is widely used following initial treatment with unfractionated heparin or low-molecular-weight heparin (5, 6). On the other hand, novel oral anticoagulants (NOACs), including a direct thrombin inhibitor (dabigatran) and factor Xa inhibitors (rivaroxaban, apixaban, and edoxaban), have recently been proven to be more effective and safer than VKAs for the prevention and treatment of stroke in patients with atrial fibrillation and deep venous thrombosis (7–9), suggesting that NOACs could be an alternative to VKAs. However, whether NOACs could replace VKAs in CVST treatment remains uncertain. Here, we conducted a systematic review between the two types of medicines for the treatment of CVST.

## Methods

### Data Sources and Selection

We comprehensively searched the PubMed database, clinical trial registries, and similar article search engines from Jan 1, 2010 to June 1, 2020 for retrospective studies, prospective studies, and randomized clinical trials (RCTs) using the terms “cerebral venous thrombosis,” “cerebral sinus thrombosis,” “cerebral sinus and dural thrombosis,” “new oral anticoagulants,” “direct oral anticoagulants,” “non-vitamin K antagonists,” “warfarin,” “phenprocoumon,” and “vitamin K antagonists.” We selected studies that compared the efficacy and safety outcomes of NOACs and VKAs. Publications in all languages and time frames were included. Duplicate articles, articles unrelated to the study aim, and articles not belonging to the article types mentioned above were excluded. The references of the selected articles were reviewed. Two reviewers screened the studies independently. Any disagreements were resolved by the adjudicating senior author.

### Data Extraction and Synthesis

All data required for the studies were extracted: identity, design, outcomes, and complications. The quality of the observational studies was then assessed by the Newcastle-Ottawa quality scale (10). The quality of RCTs was assessed by the Cochrane Risk of Bias tool (11). The outcomes extracted were efficacy and safety outcomes. The efficacy outcomes were defined according to recurrence of VTEs, death, recanalization, and functional recovery. The level of recanalization was divided into full recanalization and partial recanalization. An uninterrupted flow signal with residual luminal narrowing of <50% in all sinus previously affected was considered as full recanalization, while an uninterrupted flow signal with residual luminal narrowing of at least 50% in at least one sinus previously affected as partial recanalization (12). For functional recovery, outcomes were measured by the modified Rankin Scale (mRS), and an excellent clinical outcome was equal to mRS 0–1. The safety outcomes comprised major bleeding according to the International Society on Thrombosis and Hemostasis criteria, and overall bleeding events included other relevant bleeding events. After extracting data from the studies, we assessed the overall quality of the data by using the GRADE approach. Next, we calculated risk ratios (RRs) and the corresponding 95% confidence intervals (CIs) for individual and pooled outcomes and compared them with a fixed-effects model or random-effects model depending on between-study heterogeneity. When needed, we calculated numbers of outcome events based on event rates, sample size, and duration of follow-up. We assessed the appropriateness of pooling data across studies with the use of the Cochrane Q statistic and *I*^2^ test for heterogeneity. Publication bias was assessed by generating separate plots for each outcome.

## Results

**Figure 1:**
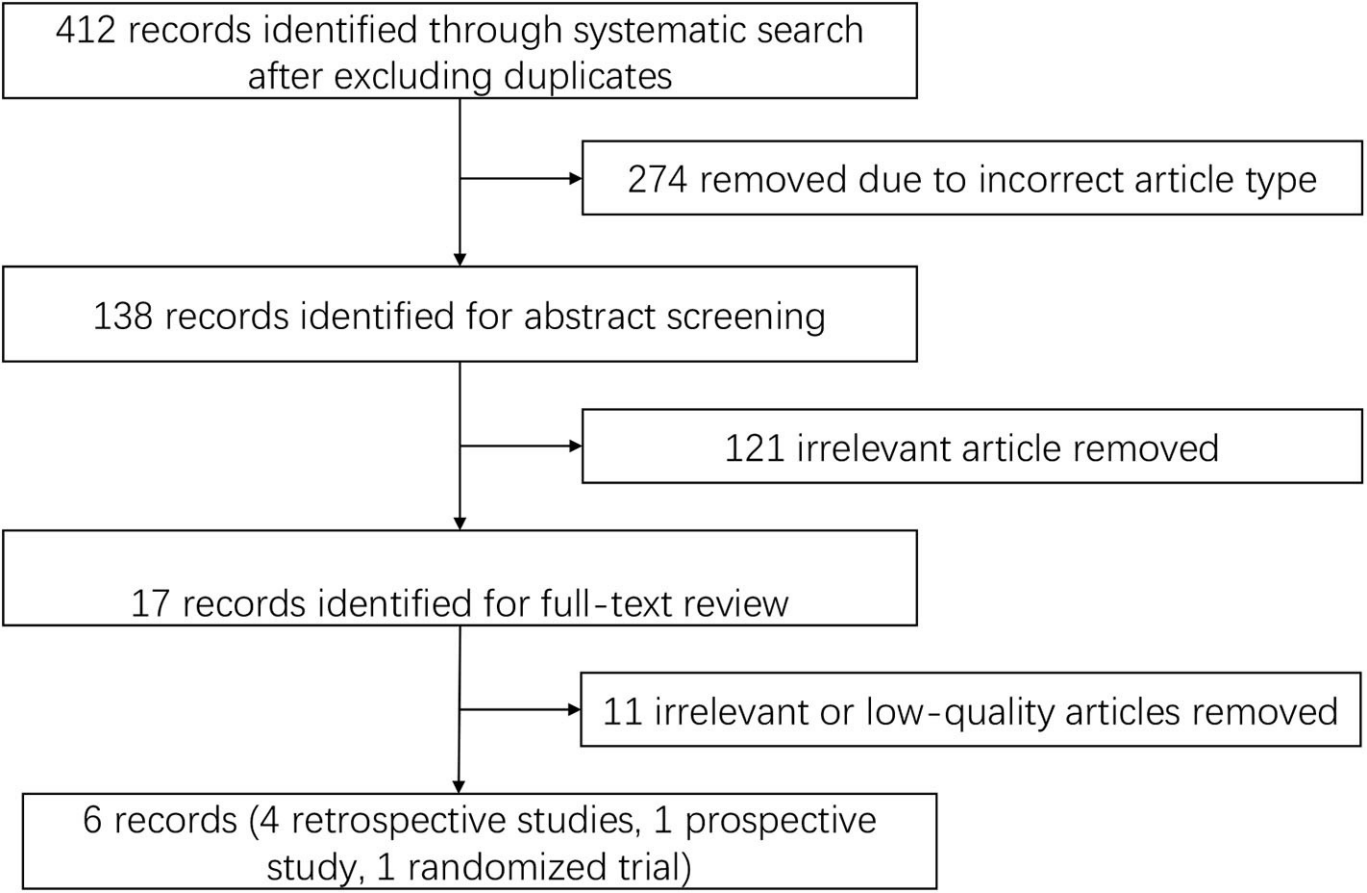
Study selection.

We identified 412 publications using the screening terms in the databases after excluding duplicates, and 274 of the records were excluded because they were not the correct article type. In further screening, 121 records were excluded after abstract reading due to irrelevant content. The remaining studies were fully reviewed by independent researchers and were excluded because of low quality and irrelevant content. Six publications were identified using the search strategy, and the search details are listed in Figure 1.

At last, six studies (four retrospective studies, one prospective study, and one RCT) including 398 patients were included (13–18). Further examination of relevant studies in the references of the included studies yielded no other studies. The study characteristics, patient selection criteria, matching process, and quality scoring for both studies are listed in Table 1. The details of the patients from both treatment groups are shown in Table 2. The RCT was assessed by the Cochrane Risk of Bias tool, and the results are shown in Table 3. Among the observational studies, four out of five were good quality (13, 14, 16, 18), and one study (17) was fair quality assessed by Newcastle-Ottawa quality scale, and the results are shown in Table 4.

**Table 1:** Basic information of included studies.

**Included study**	**Study type**	**No. of participants**	**Treatment**	**No. of women (%)**	**Mean age (years)**	**Follow-up time**	**Duration of therapy**
Wasay et al. (18)	Observational study and prospective study	111	Rivaroxaban (*n* = 36) Dabigatran (*n* = 9) Warfarin (*n* = 66)	57.7	39.3	6–13 months (median 8)	Not stated
Geisbusch et al. (13)	Observational study and retrospective study	16	Rivaroxaban (*n* = 7) Phenprocoumon (*n* = 9)	81	36 (17–75)	5–26 months (median 8)	Rivaroxaban: 6–12 months (median 8) Phenprocoumon: 7–26 months (median 9)
Mendonca et al. (14)	Observational study and retrospective study	15	Dabigatran (*n* = 11) Warfarin (*n* = 4)	80	38 (19–65)	7–35 (median 19)	Dabigatran: 3–30 months (median 6) Warfarin: not stated
Herweh et al. (17)	Observational study and retrospective study	95	NOACs (*n* = 82) Phenprocoumon (*n* = 13)	81.8	38 (17–80)	1–88 months (median 8)	1–84 months (median 7)
Lurkin et al. (16)	Observational study and retrospective study	41	Rivaroxaban (*n* = 13) Dabigatran (*n* = 2) Apixaban (*n* = 1) VKAs (*n* = 25)	55.6	44.64 (16–83)	3–11 months	NOACs: median 6 months VKA: median 8 months
Ferro et al. (15)	Randomized clinical trial	120	Dabigatran (*n* = 60) Warfarin (*n* = 60)	55	45.2	25 weeks	Dabigatran: 22.3 ± 6.16 Warfarin: 23.0 ± 5.20

**Table 2:** Baseline characteristic of included studies.

**Variable**	**No. of studies**	**No. of participants**	
		**NOACs**	**VKAs**
Women, *n* (%)	5	86 (61.9)	94 (57.3)
Median age (years)	5	40.5	43.7
Previous VTEs, *n* (%)	4	9 (6.5)	16 (19.8)
Thrombophilia, *n* (%)	4	12 (8.6)	8 (4.9)
Malignancy, *n* (%)	4	3 (2.2)	10 (6.1)
Puerperium, *n* (%)	4	12 (8.6)	8 (4.9)
Oral contraceptive, *n* (%)	5	35 (25.5)	37 (22.8)
Smoking, *n* (%)	3	12 (35.3)	12 (31.6)

**Table 3:** Quality assessment of the included RCT.

**Bias**	**Authors' judgment**	**Support for judgment**
Random sequence generation (selection bias)	Low risk	0
Allocation concealment (selection bias)	Low risk	Patients were randomized through an online telephone-guided response system.
Blinding of participants and personnel (performance bias)	High risk	The study is an open-label study.
Blinding of outcome assessment (detection bias)	Unclear risk	Insufficient evidence was presented that the assessment was independent since the study is open-label.
Incomplete outcome data (attrition bias)	Low risk	Attrition data were completely recorded in the study.
Selective reporting (reporting bias)	Low risk	No selective reporting.
Other bias	Unclear risk	Not mentioned.

**Table 4:** Quality assessment of observational studies.

**Study**	**Selection**				**Comparability**	**Outcome**			
	**Representativeness of the exposed cohort**	**Selection of the non-exposed cohort**	**Ascertainment of exposure**	**Demonstration that outcome of interest was not present at start of study**	**Comparability of cohorts on the basis of the design or analysis controlled for confounders**	**Assessment of outcome**	**Was follow-up long enough for outcomes to occur?**	**Adequacy of follow-up of cohorts**	**Final score**
Wasay et al. (18)	^*^	^*^	^*^	No star (hemorrhage events were observed at the start of the study, which is the safety outcome)	^*^	^*^	^*^	^*^	7, good quality
Geisbusch et al. (13)	^*^	^*^	^*^	No star (hemorrhage events were observed at the start of the study, which is the safety outcome)	^*^	^*^	^*^	^*^	7, good quality
Mendonca et al. (14)	^*^	^*^	^*^	No star (recurrent CVST were observed in two patients, which is the efficacy outcome)	^*^	^*^	^*^	^*^	7, good quality
Herweh (2019)	^*^	^*^	^*^	No star (hemorrhage events were observed at the start of the study, which is the safety outcome)	No star (remarkable difference between both groups)	^*^	^*^	^*^	6, fair quality
Lurkin et al. (16)	^*^	^*^	^*^	No star (not stated)	^**^	^*^	^*^	^*^	8, good quality

A study can be awarded a “*” for each item within the Selection and Exposure categories. A maximum of two “*” can be given for Comparability.

### Recurrence of VTEs or Death

**Figure 2:**
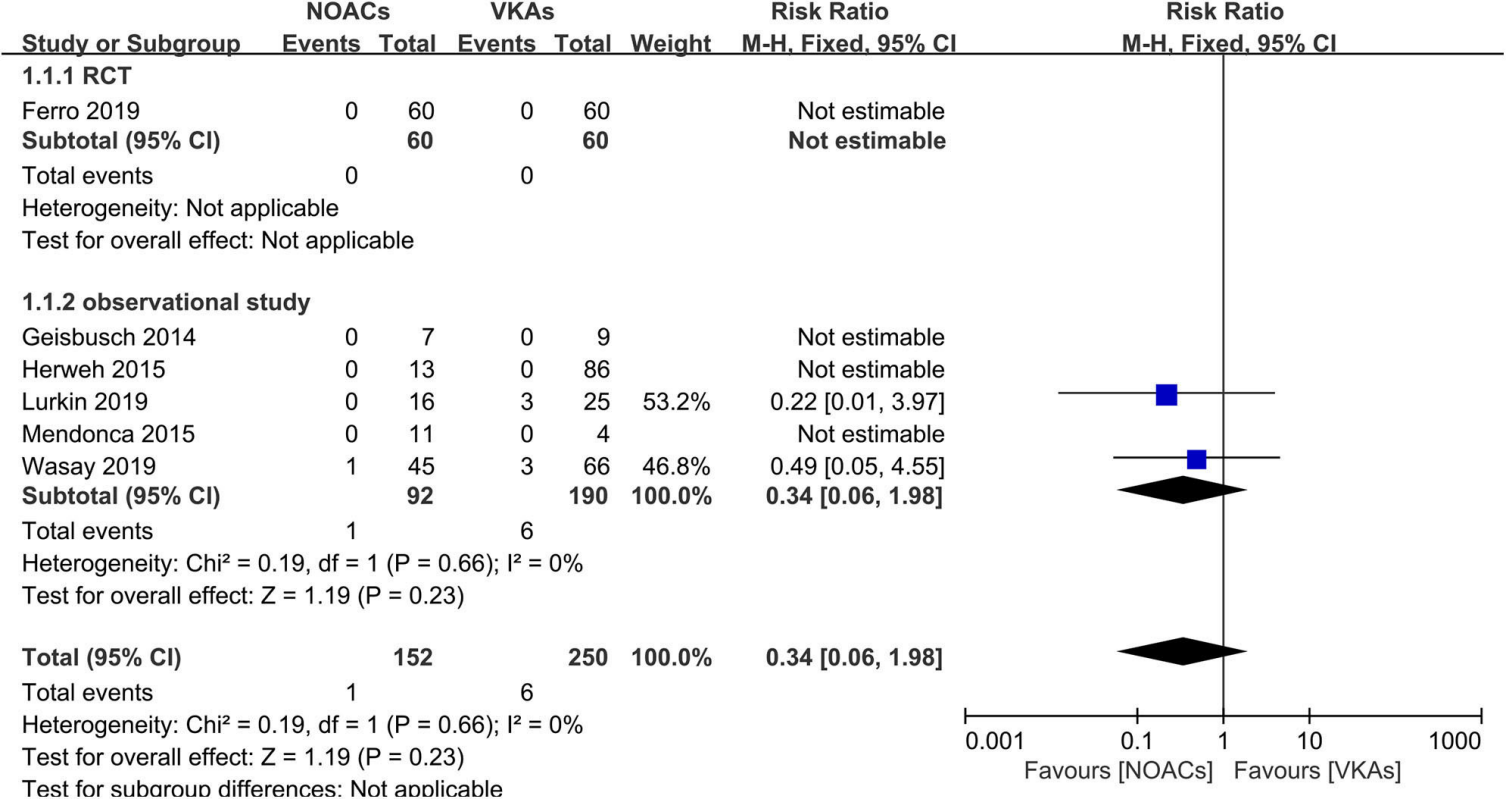
Recurrence or death between novel oral anticoagulants (NOACs) and vitamin K antagonists (VKAs).

Recurrence of VTEs or death was recorded as an outcome in every study; however, such events were reported in only two studies (16, 18). The rates of recurrence and death events were both low (Figure 2). Overall, there were only one recurrent event in the NOAC group and six in the VKA group. There was no significant difference (RR = 0.34, 95% CI 0.06–1.98) and no significant between-study heterogeneity (χ^2^ = 0.19, df = 1, *p* = 0.73, *I*^2^ = 0%) in recurrence risk between both groups.

### Recanalization

**Figure 3:**
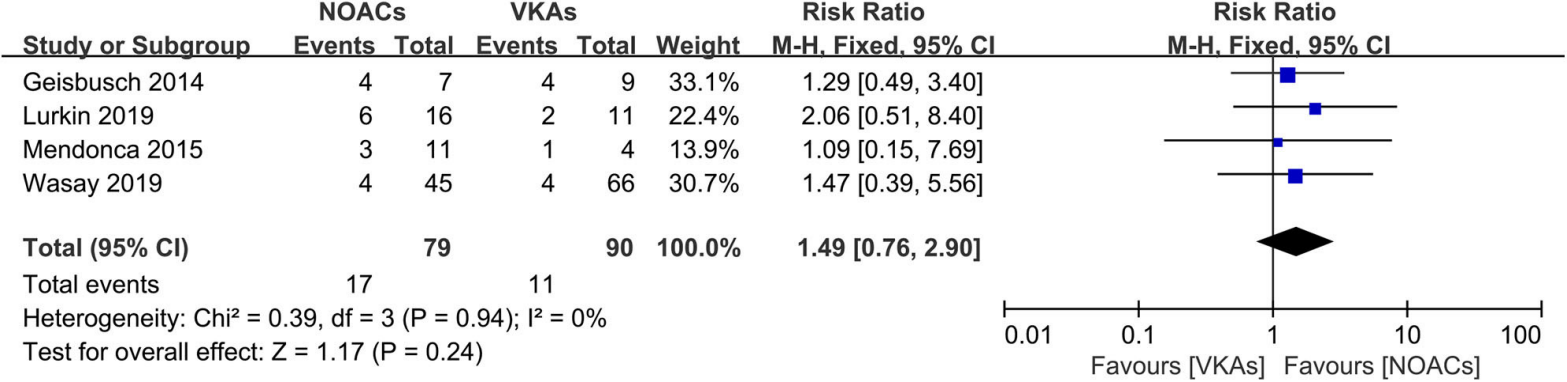
Full recanalization between NOACs and VKAs.

**Figure 4:**
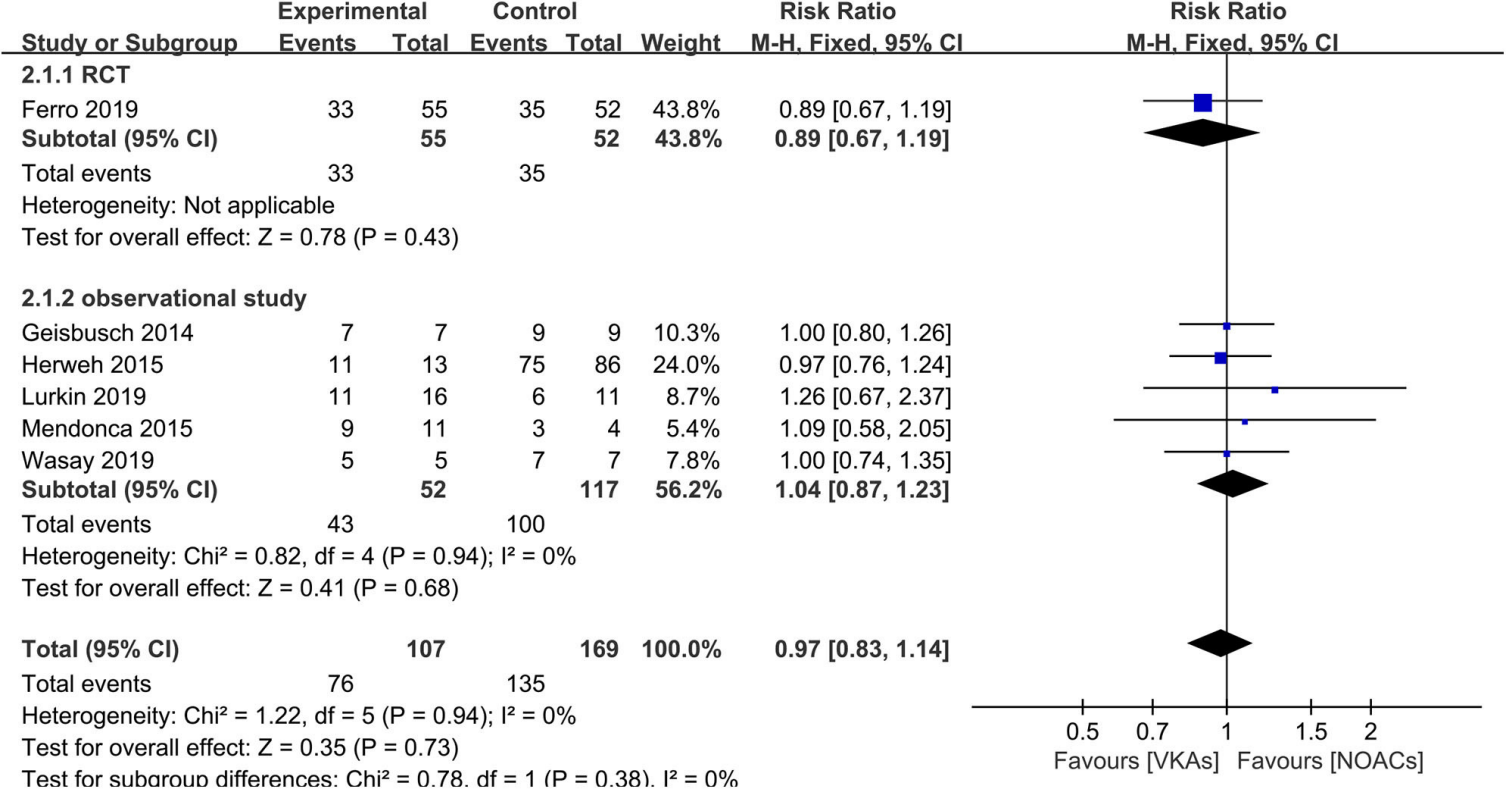
Partial recanalization between NOACs and VKAs.

The assessment of recanalization was divided into two categories (full recanalization and partial recanalization) in all included observational studies, of which two studies only reported partial recanalization. Apart from the studies described above, such outcome was recorded as an improved score of thrombus recanalization in the RCT, which we regarded as at least partial recanalization based on evidence in the study (3, 15). In the outcome of full recanalization, the data were comparable between groups (Figure 3, RR = 1.49, 95% CI 0.76–2.90), with no significant between-study heterogeneity (χ^2^ = 0.39, df = 3, *p* = 0.94, *I*^2^ = 0%). For partial recanalization, the rates in the NOAC group and VKA group were 73.5 and 80.9%, respectively. Data showed no significant difference (Figure 4, RR = 0.97, 95% CI 0.83–1.14) in the comparison of risks. No between-study heterogeneity was observed (χ^2^ = 1.22, df = 5, *p* = 0.94, *I*^2^ = 0%).

### Hemorrhage Events

**Figure 5:**
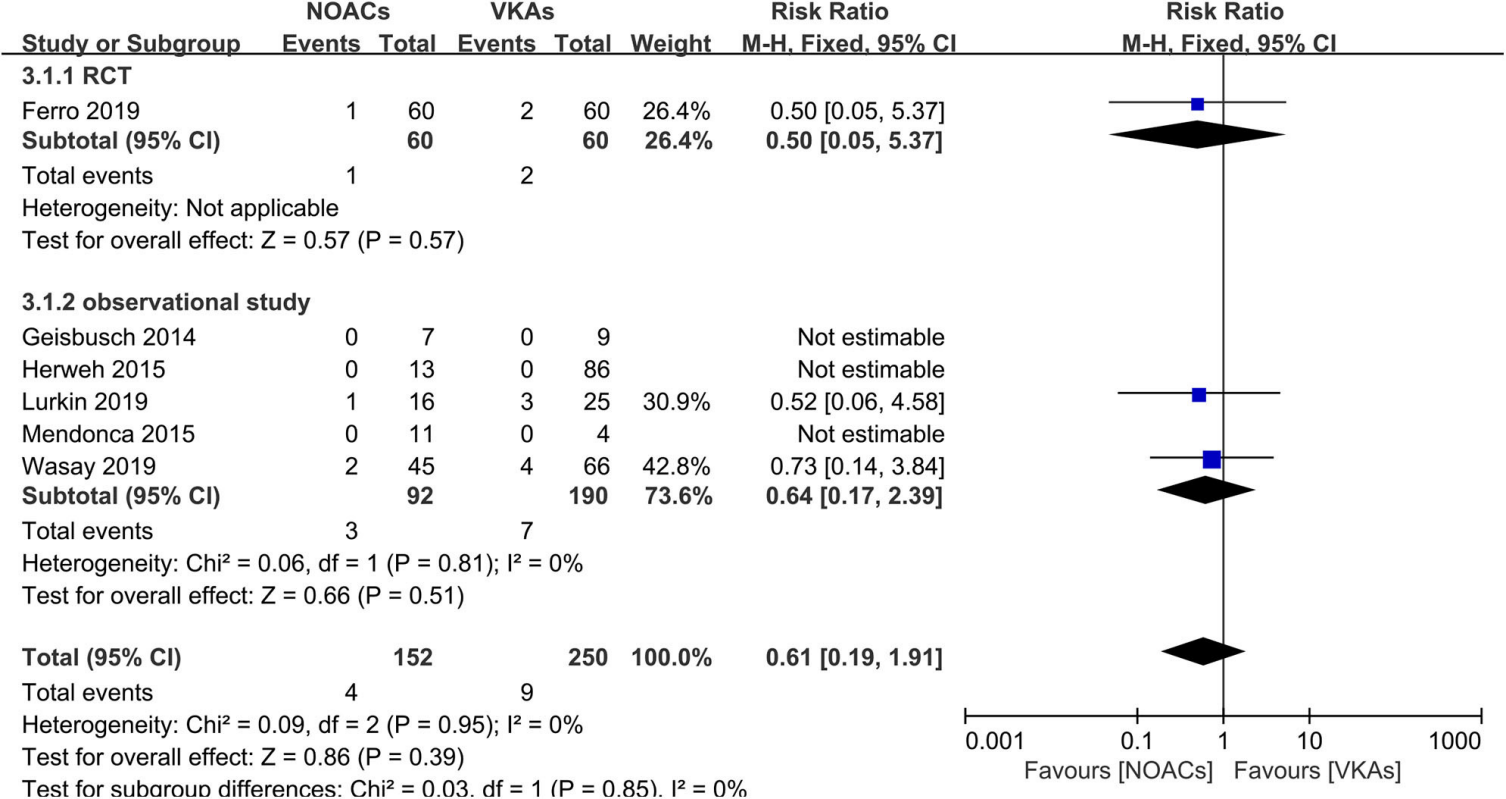
Major bleeding events between NOACs and VKAs.

**Figure 6:**
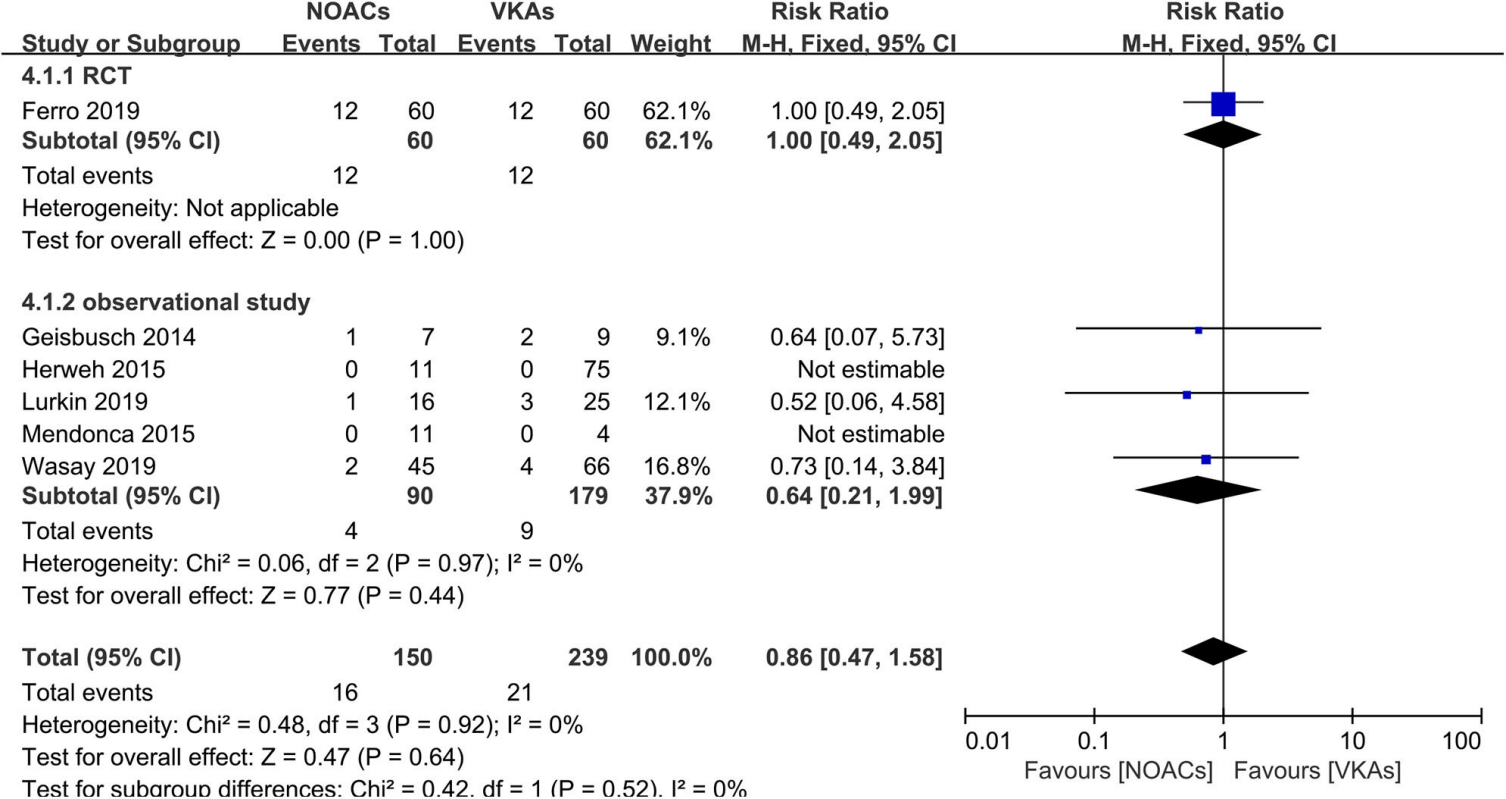
Overall bleeding events between NOACs and VKAs.

Hemorrhage events were also recorded as an outcome in every study (Figure 5). The rates of major hemorrhage events were relatively low in both groups (2.6 and 3.6%), and there was no significant difference (RR = 0.61, 95% CI 0.19–1.91) and no significant between-study heterogeneity (χ^2^ = 0.03, df = 1, *p* = 0.85, *I*^2^ = 0%) in the assessment of the risks in both groups. Moreover, in the comparison of overall bleeding events that included other bleeding events, such as minor bleeding and clinically relevant bleeding events, the data showed no statistical significance (RR = 0.86, 95% CI 0.47–1.58) with no significant between-study heterogeneity (Figure 6, χ^2^ = 0.48, df = 3, *p* = 0.92, *I*^2^ = 0%).

### Excellent Clinical Outcome

**Figure 7:**
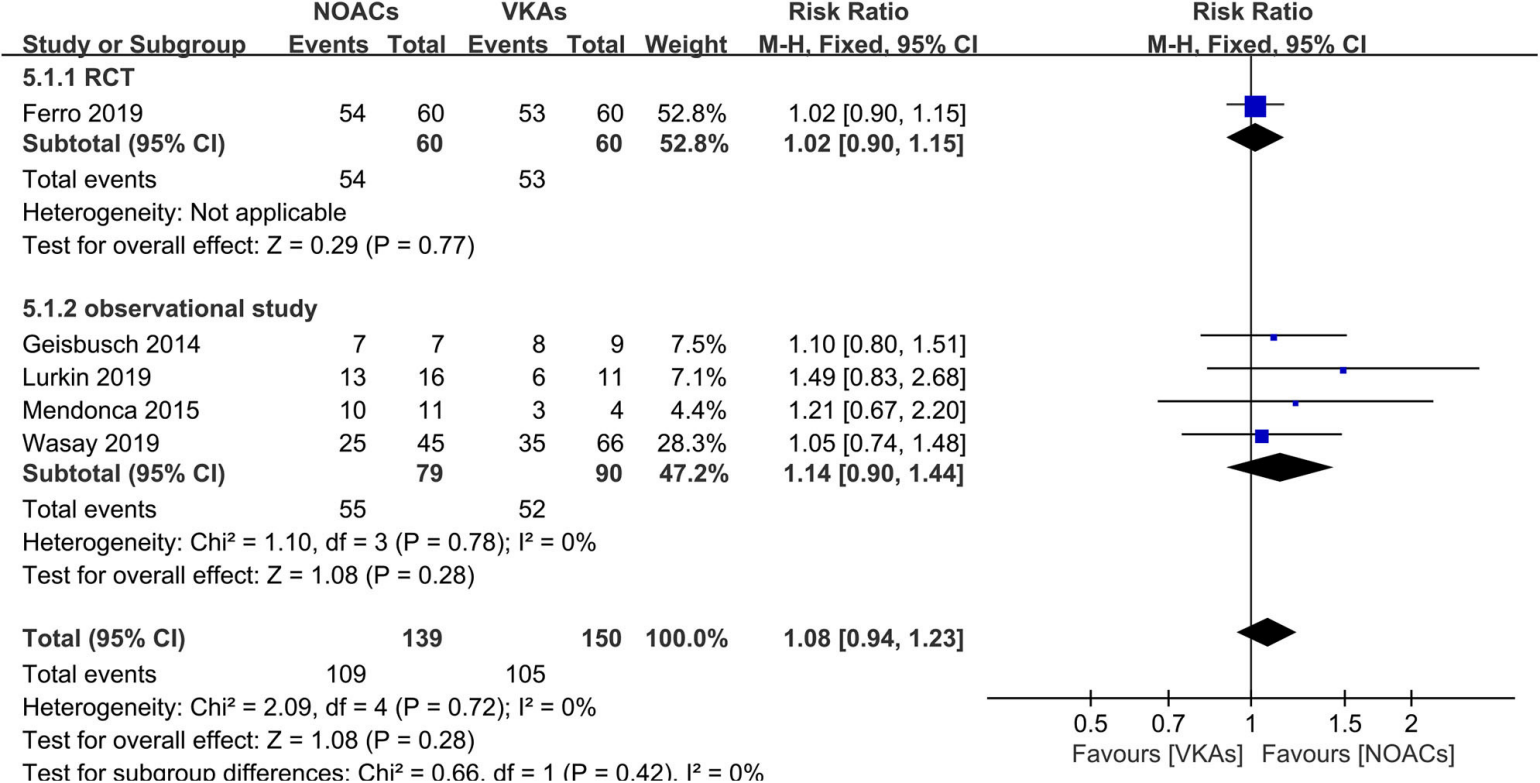
Excellent clinical outcome between NOACs and VKAs.

All six studies conducted a separate comparison of NOACs and VKAs in regard to mRS. A study (17) did not conduct separate statistical analyses between groups. In total, 78.4% (109/139) of patients in the NOAC group and 70.0% (105/150) of patients in the VKA group achieved excellent clinical outcomes after anticoagulant therapy (Figure 7). There was no significant difference (RR = 1.08, 95% CI 0.94–1.23) in the rates and no between-study heterogeneity (χ^2^ = 0.66, df = 1, *p* = 0.42, *I*^2^ = 0%).

### Subgroup Comparison of NOACs and VKAs

#### Dabigatran vs. VKAs

**Figure 8:**
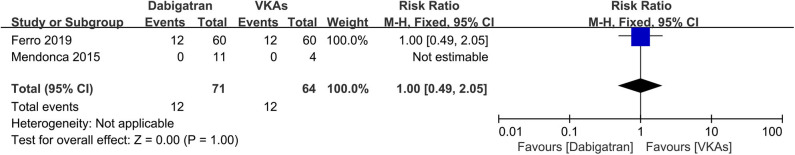
Bleeding events between dabigatran and VKAs.

**Figure 9:**
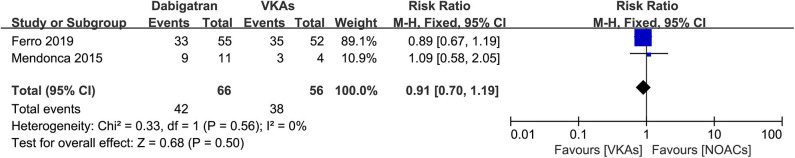
Recanalization between dabigatran and VKAs.

**Figure 10:**
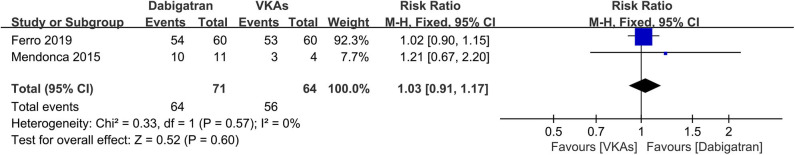
Excellent clinical outcome between dabigatran and VKAs.

Of the included studies, two out of six (14, 15) conducted an independent comparison between a certain kind of NOAC, dabigatran, and VKAs. We compared similar outcomes (bleeding events, recanalization, and excellent clinical outcome) as described above. As a result, the rates of bleeding events, recanalization, and excellent clinical outcome were comparable between patients in both groups (Figures 8–10), and the RRs of bleeding events, recanalization, and excellent clinical outcome were 1.00 (95% CI 0.49–2.05), 0.91 (95% CI 0.70–1.19), and 1.03 (95% CI 0.91–1.17), respectively. No between-study heterogeneity was observed.

#### NOACs vs. Phenprocoumon

**Figure 11:**
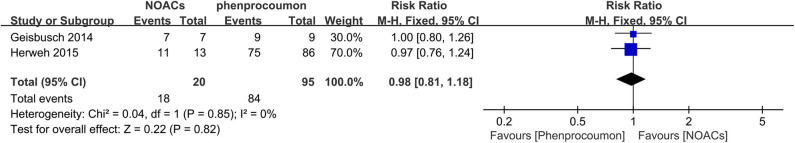
Recanalization between NOACs and phenprocoumon.

Two studies (13, 17) conducted a direct comparison between NOACs and phenprocoumon, and outcomes were comparable for at least partial recanalization (Figure 11). The rates of partial recanalization between NOACs and phenprocoumon were not significantly different (RR = 0.98, 95% CI 0.81–1.18), with no between-study heterogeneity (χ^2^ = 0.04, df = 1, *p* = 0.85, *I*^2^ = 0%).

#### NOACs vs. Warfarin

**Figure 12:**
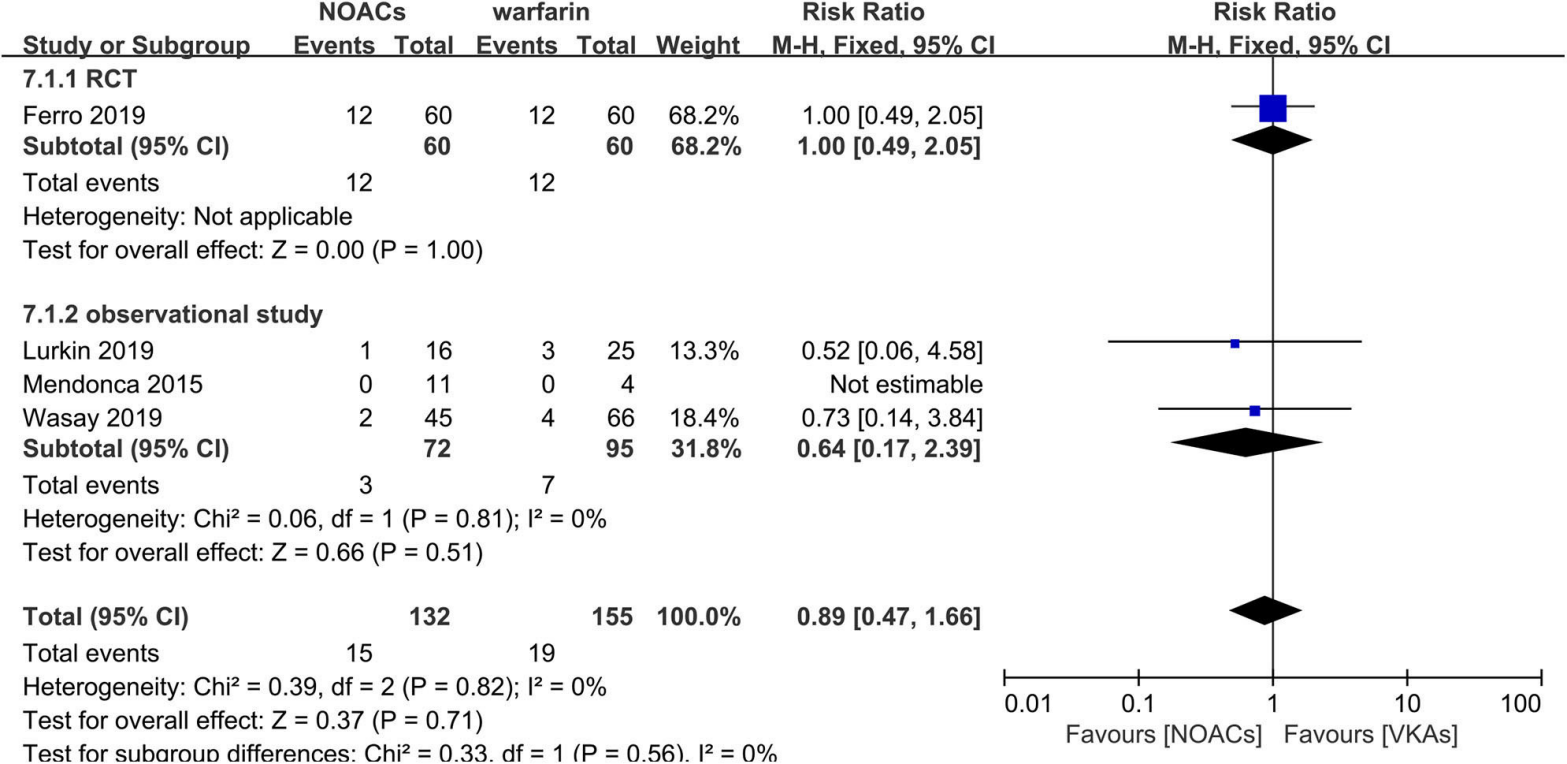
Bleeding events between NOACs and warfarin.

**Figure 13:**
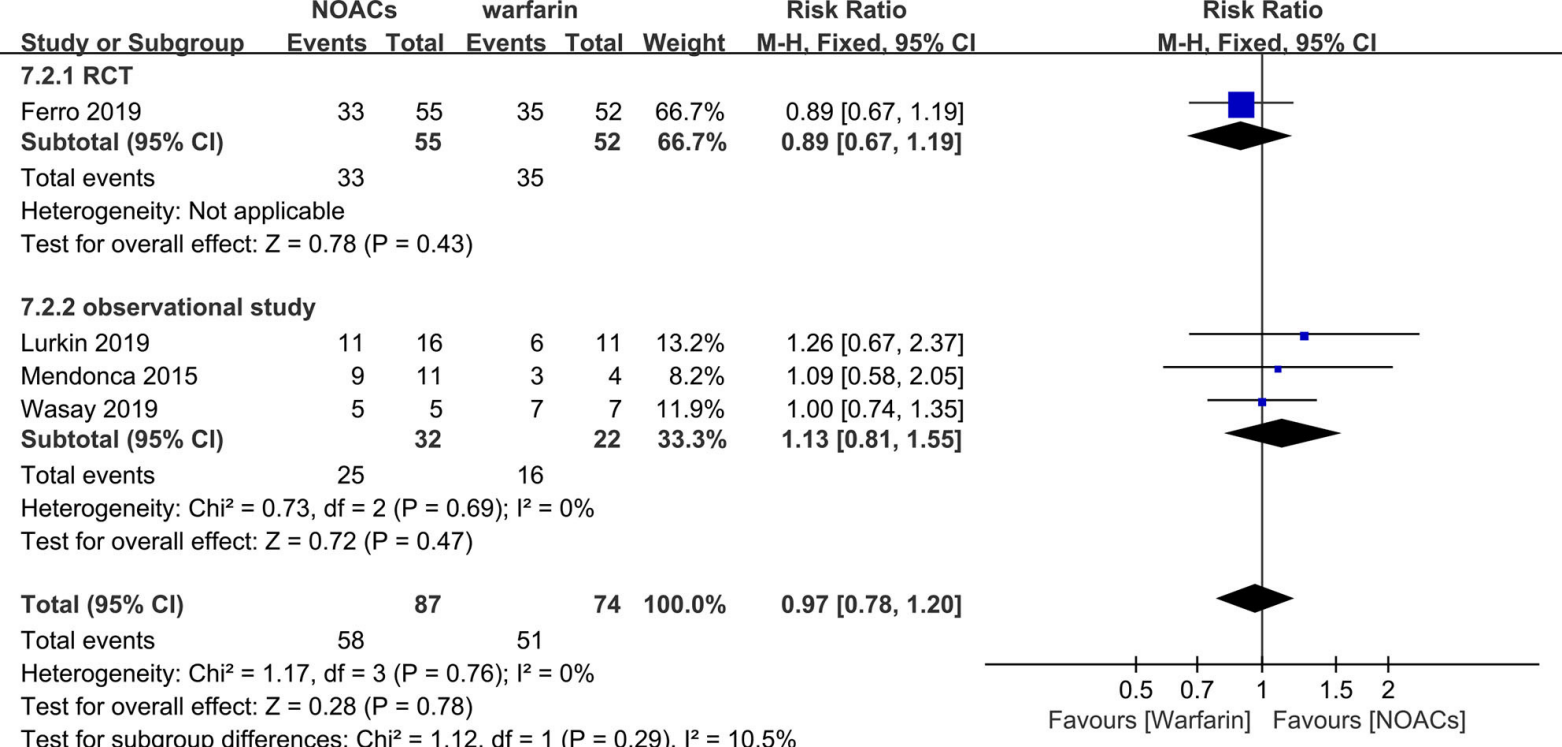
Recanalization between NOACs and warfarin.

**Figure 14:**
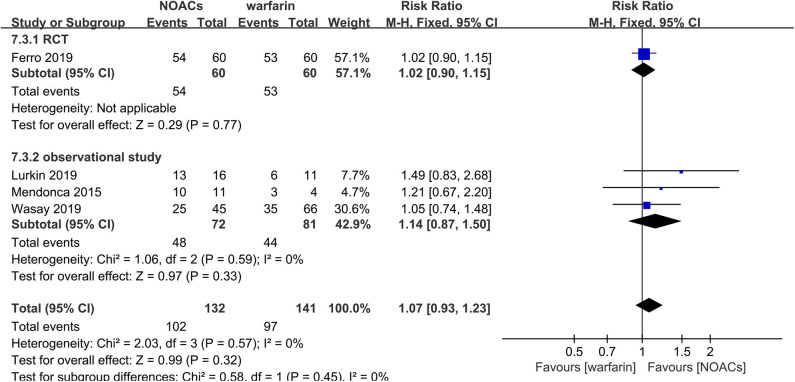
Excellent clinical outcome between NOACs and warfarin.

Direct comparison between NOACs and another VKA, warfarin, was performed by four studies (14–16, 18). Recanalization, hemorrhage events, and functional recovery were comparable (Figures 12–14). No significant difference was observed in the outcomes above, and the RRs for recanalization, hemorrhage events, and functional recovery were 0.97 (95% CI 0.78–1.20), 0.89 (95% CI 0.47–1.66), and 1.07 (95% CI 0.93–1.23), respectively, with no between-study heterogeneity.

## Discussion

In the present comparison between NOACs and VKAs for the treatment of CVST, we found that NOACs may be as effective and safe as VKAs in regarding thrombotic and hemorrhagic events with limited RCT data available. Nonetheless, there was no significant difference between the two treatments. In conclusion, the application of NOACs for CVST is similar to that of VKAs in terms of efficacy and safety.

To prevent CVST, long-lasting anticoagulants, mainly VKAs, were routinely applied after bridging with heparin. It has been proven that NOACs have advantages over VKAs since they were introduced in the last decade. The major advantage of NOACs is that they target a single site in the coagulation cascade rather than multiple sites as VKAs do, which makes NOACs more potent for the prevention of hemorrhage. Moreover, NOACs have advantages such as not requiring international normalized ration monitoring, rapid onset, and shorter half-lives. Previous studies have indicated that NOACs are more effective and safer than warfarin for stroke prevention in patients with atrial fibrillation (7), especially in Asian populations (8), with a reduction in bleeding events. In the context of symptomatic deep vein thrombosis, NOACs are not inferior to warfarin on efficacy and dramatically reduce hemorrhagic events (9). NOACs could be guideline alternatives to VKAs for these thromboembolic diseases. However, whether NOACs could replace warfarin in CVST treatment remains unclear.

We conducted a comprehensive comparison of efficacy and safety between NOACs and VKAs based on the current evidence. After extracting and analyzing the data from six studies covering 398 patients, we found no significant difference in the efficacy outcomes of recurrence or death, recanalization, and clinical recovery or safety outcomes. In the subgroup comparisons of NOACs or VKAs (dabigatran vs. VKAs, NOACs vs. phenprocoumon, and NOACs vs. warfarin), no significant difference was found between patients in outcomes. Altogether, we revealed that NOACs is similar to VKAs for CVST treatment in terms of efficacy and safety based on the existing evidence, suggesting that NOACs could be potential alternatives to VKAs. At the meantime, a trend toward reducing hemorrhage events was also observed. The present results were similar to a published report by Lee et al. (19). In our study, however, we included a new high-quality study and excluded a low-quality retrospective study (20) that was included by Lee. Moreover, we performed a comprehensive subgroup comparison of different NOACs and VKAs (dabigatran vs. VKAs, NOACs vs. phenprocoumon, and NOACs vs. warfarin), further evaluating the efficacy and safety of the two types of medicines in detail.

Since CVST is a rare type of stroke with relatively low morbidity, it is not surprising for the lack of high-quality studies due to the difficulty to collect enough cases in the context of CVST therapy. Therefore, the major limitation of our study was a relatively small number of studies and patients included, mainly with observational studies except one RCT. Thus, the ability to identify the difference of efficacy and safety between NOACs and VKAs was not strong enough since there are limited RCT data available. Meanwhile, studies comparing other specific NOACs (e.g., apixaban and edoxaban) have not been available yet. Noteworthily, several RCTs (NCT03747081, NCT03178864, and NCT04569279) comparing NOACs (rivaroxaban) with VKAs (warfarin) are in progress. These encouraging studies would undoubtedly more helpful to clarify the issue.

## Data Availability Statement

The original contributions presented in the study are included in the article/supplementary materials, further inquiries can be directed to the corresponding author/s.

## Author Contributions

All authors listed have made a substantial, direct and intellectual contribution to the work, and approved it for publication.

## Conflict of Interest

The authors declare that the research was conducted in the absence of any commercial or financial relationships that could be construed as a potential conflict of interest.
